# Characteristics of the needs of older people in Kazakhstan: a cross-sectional muliticentre study

**DOI:** 10.3389/fpubh.2025.1702417

**Published:** 2025-11-25

**Authors:** Lyudmila Yermukhanova, Kerbez Kimatova, Aleksandra Suwalska, Ian Philp, Marzena Dworacka, Perizat Aitmaganbet, Saule Tazhbenova, Maiya Taushanova, Gulnara Kurmanalina, Dorota Talarska, Katarzyna Wieczorowska-Tobis, Slawomir Tobis

**Affiliations:** 1 Department of Public Health and Healthcare, Marat Ospanov West Kazakhstan Medical University, Aktobe, Kazakhstan; 2 Department of Mental Health, Chair of Psychiatry, Poznan University of Medical Sciences, Poznań, Poland; 3 Centre for Health Services Studies, University of Kent, Canterbury, Kent, United Kingdom; 4 Department of Pharmacology, Poznan University of Medical Sciences, Poznań, Poland; 5 Center for the Improvement of Nursing, Department of Public Health and Healthcare, Marat Ospanov West Kazakhstan Medical University, Aktobe, Kazakhstan; 6 Department of Internal Medicine №2, Marat Ospanov West Kazakhstan Medical University, Aktobe, Kazakhstan; 7 Department of Preventive Medicine, Poznan University of Medical Sciences, Poznań, Poland; 8 Geriatrics Unit, Chair of Palliative Medicine, Poznan University of Medical Sciences, Poznań, Poland; 9 Department of Occupational Therapy, Poznan University of Medical Sciences, Poznań, Poland

**Keywords:** older adults, unmet needs, cluster analysis, EASYCare, Central Asia

## Abstract

**Objectives:**

Shrinking caregiver workforces and rising demand among the oldest age groups necessitate urgent healthcare reforms. In Kazakhstan, high fertility sustains population growth, though care pressures are increasing as the share of older adults rises. Family, mostly women, provide care, and societal changes are reshaping traditional caregiving roles. The aim of the study was to assess the health and social needs of older adults in Kazakhstan and uncover their groupings.

**Methods:**

A cross-sectional multicentre study (2020–2021) in four Kazakhstani cities surveyed outpatients aged ≥65 years with the EASYCare Standard 2010 questionnaire. Functional independence, risk of care breakdown, and falls were measured, and *k*-means clustering identified need profiles. Linguistic diversity required Russian-language assessments.

**Results:**

Among 452 participants (mean age 70.7 ± 5.7 years), three clusters emerged. Lower unmet needs correlated with higher education and less caregiver support; higher needs were linked to lower education and frequent informal care. Fewer than 1% used formal services.

**Conclusion:**

Unmet needs among older Kazakhs are linked to lower education and informal care. Expanding formal care and targeted interventions are essential for supporting the ageing population’s varied needs.

## Introduction

The global demographic shift toward an ageing population, as evidenced by increasing life expectancy at birth, has been a defining characteristic of recent decades. The most demographically advanced nations, including Japan and Western European countries, now report that over 20% of their populations are aged 65 years or more, whilst experiencing negative natural population growth (1, 2). This demographic transition necessitates urgent and comprehensive reforms of healthcare systems to address the complex and evolving needs of rapidly expanding older cohorts (3). In these contexts, workforce availability—including that of professional caregivers—is diminishing, while demand for support among the oldest age groups continues to increase (4).

The demographic landscape in Kazakhstan presents a distinctly different profile from that of its Western counterparts (5). Although the number of older people is indeed increasing, fertility rates remain comparatively high, and projections indicate that Kazakhstan’s population will continue to grow over the next three decades (6). Nevertheless, the growing proportion of older individuals generates increased demand for support services and also raises concerns regarding the adequacy of human resources for care provision (7).

### Care provision and family structure

In Kazakhstan, as throughout Central Asia, care for individuals requiring support is predominantly delivered by family members (8). The Constitution of the Republic of Kazakhstan explicitly mandates that able-bodied children bear responsibility for the care of their disabled older parents (9, 10).

Although it is frequently emphasised that interpersonal relationships within contemporary Kazakh families remain strongly influenced by national culture and traditions, there is a discernible trend—mirroring developments observed in many countries globally—toward transforming extended, multigenerational family structures into nuclear family units (11). This demographic shift, combined with increasing participation of older women in the workforce, complicates the daily provision of care, as caregiving responsibilities continue to be borne predominantly by women (8). Although comprehensive data on the impact of daily care burdens on family caregivers remains limited throughout Central Asian countries, research addressing these issues is already being conducted in Kazakhstan (12).

The distinctive role of informal caregivers within Kazakhstan’s care system is underscored by the finding that, when older individuals require physical support, fewer than 1% seek assistance from formal services (13). The same research, conducted within the geriatric population in Almaty, Kazakhstan’s largest city, revealed that approximately one in five older individuals surveyed required some form of assistance (13). The infrequent recourse to formal services stems not only from enduring cultural traditions but also from the limited availability of such services, particularly in rural areas (14).

### Needs assessment and language considerations

The development of appropriate services must respond to established needs. To our knowledge, comprehensive studies examining the medical and social needs of older people have not been undertaken in Kazakhstan or other Central Asian countries [a study of needs, regarding the city of Almaty, was recently conducted using the Camberwell Assessment of Needs questionnaire (15)]. Addressing this research gap, our team developed and validated a Kazakh-language version of the internationally recognised EASYCare Standard 2010 questionnaire (ECQ) (16). The ECQ is a comprehensive geriatric assessment instrument designed for evaluating physical, mental and social functioning, as well as unmet health and social needs among older people (17). The instrument incorporates elements from established measures, including basic activities of daily living, selected instrumental activities of daily living, safety assessments, accommodation and financial evaluations, and well-being indicators. Evidence supports its use for individual needs assessment, with good validity and positive endorsements regarding acceptability from both older people and practitioners (18). Moreover, Marques et al. provided a mapping between the EC domains and the WHO ICF framework (19).

Kazakhstan’s multilingual character presents certain challenges for needs assessment. According to the 2021 National Population Census, Kazakhstan is a linguistically diverse country, with 44.9% of residents possessing knowledge of two languages and 28.6% knowing three languages (20, 21). Although 80.1% of the total population demonstrates proficiency in Kazakh, the state language, among older people (9.11% of the total population), most use the Russian language, with only 64.4% of those aged 65–69 and 56.9% of those aged 70 and above knowing Kazakh (20). These demographic patterns reflect the linguistic socialisation experiences of older cohorts who received their education during the Soviet period, when Russian served as the primary medium of communication. When surveys are administered exclusively in languages with which potential respondents lack proficiency, the likelihood of participation diminishes substantially; thus, offering appropriate language options can facilitate participation and improve data quality (22). Therefore, we conducted our study with a Russian-language version of the tool, allowing older people to use their preferred language version to optimise the accessibility of the questionnaire.

The aim of this paper is to present the results of the diagnosis of health and social needs conducted in a large group of Kazakhstan residents who chose the Russian-language version of this tool. Our study fills a gap in this field and enables the planning of tailored interventions.

## Methods

This cross-sectional study was conducted in Kazakhstan between 2020 and 2021 using the ECQ. Data collection was performed by trained research staff. The study was funded by the Science Committee of the Ministry of Education and Science of the Republic of Kazakhstan (AP09562783) and received approval from the bioethical committee of the West Kazakhstan Marat Ospanov Medical University, Aktobe, Kazakhstan (October 14, 2020; No. 8).

### Participants

Eligible participants were individuals aged 65 years and older who were attending outpatient clinics and provided written informed consent. Inclusion criteria required full verbal and logical contact and the absence of a diagnosis of cognitive impairment. Recruitment was facilitated by general practitioners, social workers, and nurses using a convenience sampling approach. After the subjects consented to participate, meetings were organised either at their homes or at outpatient clinics as convenient. The study was conducted in four centres: Aktobe and Uralsk (western Kazakhstan), and Shymkent and Kyzylorda (southern Kazakhstan). Twenty-eight individuals declined participation, and a further 26 were excluded due to being underage. Ultimately, 452 participants were included in the analysis: 100 in Aktobe, 92 in Uralsk, 103 in Kyzylorda, and 157 in Shymkent. All participants of the current study declared Russian as their first language.

### Procedure and instruments

The English version of the EASYCare Standard 2010 questionnaire was translated and validated in Russian, demonstrating good psychometric properties (23).

The ECQ comprises 49 items across seven domains, assessing physical, mental, and social needs:

- *Seeing, hearing, and communicating* (4 items)
- *Looking after yourself* (13 items)
- *Getting around* (8 items)
- *Safety* (5 items)
- *Accommodation and finances* (3 items)
- *Staying healthy* (7 items)
- *Mental health and well-being* (9 items)

From the questionnaire data, three summarising indices were calculated for each participant:

1. *Independence score* (0–100 points): Reflects functional capacity, with higher scores indicating greater dependency.
2. *Risk of breakdown in care* (0–12 points): Indicates the risk of hospitalisation, with higher scores denoting increased risk.
3. *Risk of falls* (0–8 points): A score above 2 suggests an elevated risk of falling.

The interpretation and calculation of these indices have been described in detail elsewhere (24).

### Statistical analysis

Statistical analyses were performed using STATISTICA 13.3 software (TIBCO Software, Poland). The normality of distributions was assessed with the Shapiro–Wilk test. Results are presented as means and standard deviations, as well as medians and interquartile ranges (25th and 75th percentiles), due to the non-normal distribution of most variables.

Differences between clusters were assessed using the χ^2^ test for categorical variables and analysis of variance (Kruskal–Wallis test with Dunn’s *post-hoc* test) for continuous variables. A *p*-value of <0.05 was considered statistically significant.

### Finding the optimum number of clusters

To determine the optimum parameters for clustering, first an Elbow plot was created, relating the Sum of Squared Errors (SSE) to the underlying number of clusters (*k*).

The Elbow plot (SSE vs*. k*) reveals a distinct inflexion point at *k* = 3: the transition from *k* = 2 to *k* = 3 results in a substantial decrease in SSE (−432.9), whereas a further increase in the number of clusters to *k* = 4 yields only a comparatively modest additional error reduction (−113.4). Therefore, *k* = 3 represents an optimal balance between model simplicity and goodness of fit, justifying the selection of three clusters for subsequent analyses.

Next, the Silhouette analysis was performed.

The Silhouette coefficient peaks at *k* = 3 (0.38), indicating the best cluster separation. Consistently, the Calinski–Harabasz index (CH) is highest at *k* = 3 (269.85), while the Davies–Bouldin index (DB) is lowest at *k* = 3 (0.99). For *k* ≥ 4, Silhouette declines (0.30–0.29), CH decreases (253.23 → 227.10), and DB does not improve (1.07–1.03). Taken together, these three criteria clearly support selecting *k* = 3 as the optimal balance of compactness and separation.

Stability analyses demonstrate high reproducibility of the *k* = 3 solution. Across different random initialisations, the median ARI was 0.84 (min 0.69), indicating that cluster assignments are largely independent of initialisation. Bootstrap resampling against a reference model yielded a median ARI = 0.7 (min 0.68), evidencing robustness of the cluster structure to sampling variability. Taken together, these findings confirm that *k* = 3 is not only optimal according to separation metrics but also stable and replicable; occasional lower ARI values are incidental and do not undermine the overall clustering pattern.

### The clustering algorithm

**Figure 1:**
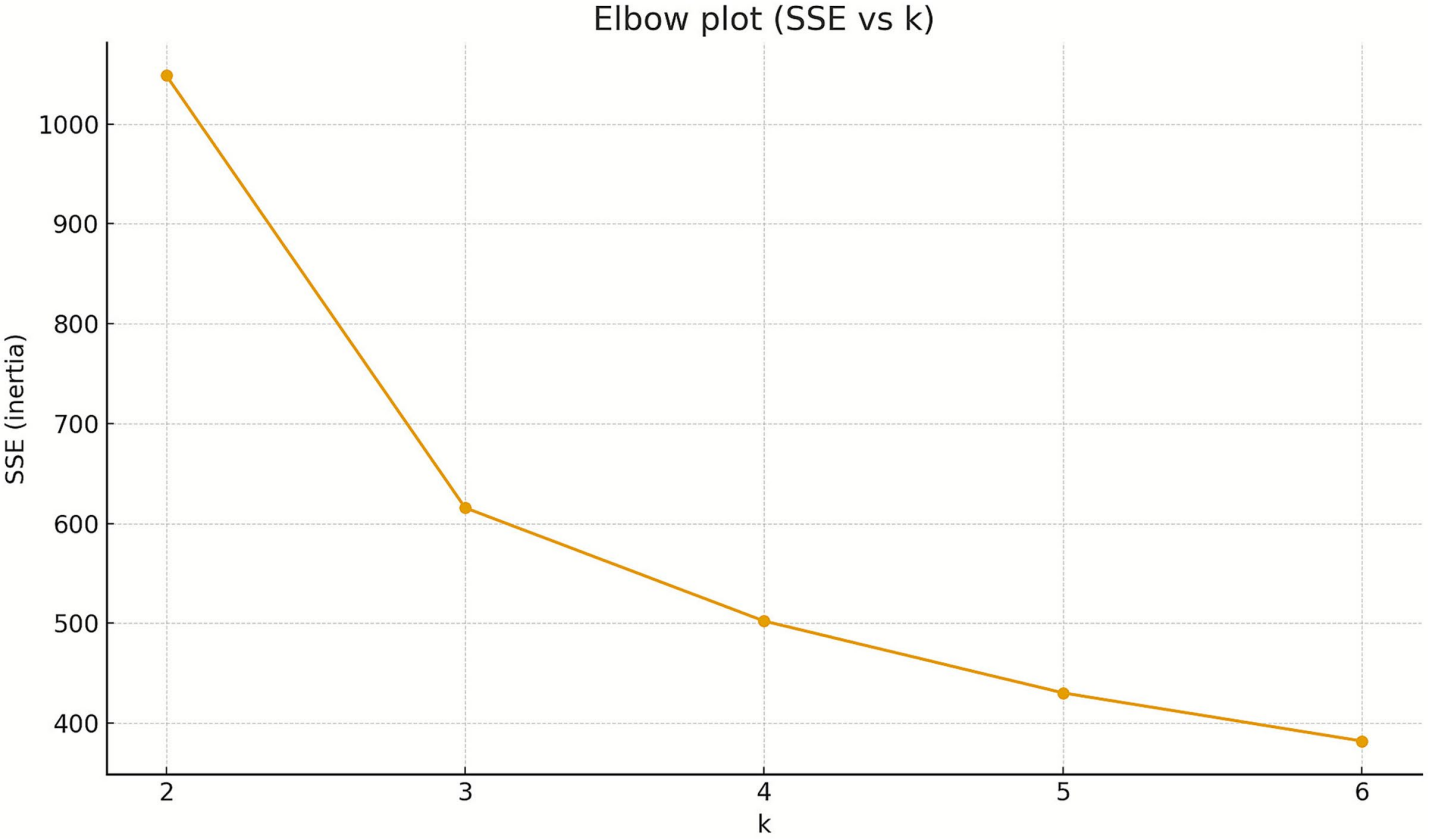
Determining the optimum number of clusters: the Elbow plot.

**Figure 2:**
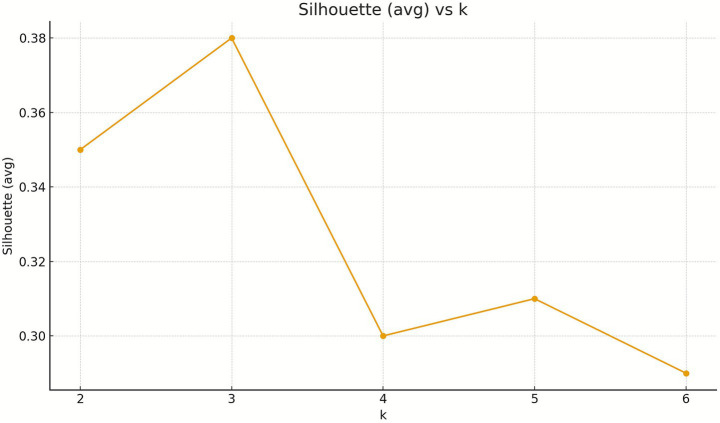
Determining the optimum number of clusters: the Silhouette coefficient plot.

We compared three clustering approaches: *K*-means, *K*-medoids (PAM), and Gaussian Mixture Models (GMM). After *z*-score standardisation, *K*-means achieved the highest Silhouette at *k* = 3 (0.38) and showed a clear inertia elbow between *k* = 2 and *k* = 3, indicating the best trade-off between within-cluster compactness and between-cluster separation. *K-*medoids reached its best Silhouette at *k* = 2 (0.36), consistent with a coarser, medoid-based partition. GMM with full covariance minimised BIC at *k* = 5, hinting at finer, elliptical sub-groups, although this did not translate into superior separation (silhouette) over the *K*-means *k* = 3 solution (Figures 1, 2).

Agreement of labels across algorithms at *k* = 3 was moderate: ARI (*K*-means vs. *K*-medoids) ≈ 0.56, ARI (*K*-means vs. GMM) = 0.33, ARI (*K*-medoids vs. GMM) = 0.27. These results suggest that while all methods capture a common dominant structure, they differ in boundary details (e.g., spherical vs. elliptical assumptions, medoid sensitivity). Considering the best separation by Silhouette for *k* = 3 with *K-*means, the clear inertia elbow, the high stability of the *k* = 3 solution (median ARI 0.84–0.87 across seeds and bootstrap; see stability section), and the parsimony and interpretability of three profiles, we selected *K*-means with *k* = 3 as the primary solution.

### *K*-means cluster analysis

*K*-means clustering was employed to identify natural groupings within the heterogeneous study population, using the three summary indices as variables. As these indices are measured on different scales, all scores were standardised to z-scores prior to cluster analysis. *K*-means clustering is an unsupervised learning technique employed to identify latent structures within heterogeneous datasets. The algorithm initiates by allocating observations randomly to a predefined number of clusters. For each cluster, a centroid is calculated as the mean of the constituent observations. Data points are subsequently reassigned to the cluster whose centroid lies at the minimal distance from them. The recomputation of centroids and reassignment of observations is performed iteratively until stability is achieved, that is, when successive iterations yield no alteration in cluster memberships (Table 1).

**Table 1:** Stability across seeds.

Method	Runs	ARI comparison	ARI (min)	ARI (median)	ARI (mean)
Various initialisations	6	15	0.69	0.84	0.84
Bootstrap	8	8	0.68	0.87	0.84

## Results

### Sample characteristics

The study group comprised 452 individuals, with a mean age of 70.7 ± 5.7 years (median: 69 years, interquartile range: 67–73 years), including 101 participants aged 75 years or older (22.3%). There were 189 men (41.8%) in the sample. Detailed demographic characteristics by gender are presented in Table 2.

**Table 2:** Demographic characteristics of the study group by gender.

Studied parameter		Total (*n* = 452)	Females (*n* = 263)	Males (*n* = 189)
Age (years)	65–74	351 (77.6%)	201 (76.4%)	150 (79.4%)
	75+	101 (22.4%)	62 (23.6%)	39 (20.6%)
Residence area	Rural	18 (4.0%)	8 (3.0%)	10 (5.3%)
	Urban	434 (96.0%)	255 (97.0%)	179 (94.7%)
Marital status	Single	75 (16.6%)	97 (36.9)	64 (33.9%)
	Married	377 (83.4%)	166 (63.1)	125 (66.1%) ***p* < 0.05**
Living arrangements	Alone	79 (17.5%)	38 (14.4%)	41 (21.7%)
	With spouse	140 (31.0%)	76 (28.9%)	64 (33.8%)
	With extended family	233 (51.5%)	149 (56.6%)	84 (44.4%) ***p* < 0.01**
Education	Primary	123 (27.2%)	75 (28.5%)	48 (25.4%)
	Secondary	172 (38.0%)	109 (41.4%)	63 (33.3%)
	Higher education	157 (34.7%)	79 (30.0%)	78 (41.3%) ***p* < 0.05**
Financial situation	Not enough to make ends meet	139 (30.7%)	86 (32.7%)	53 (28.0%)
	At least enough to make ends meet	313 (69.3%)	177 (67.3%)	136 (72.0%)
Are you a carer for someone?	Yes	116 (25.7%)	66 (25.1%)	50 (26.5%)
	No	336 (74.3%)	197 (74.9%)	139 (73.5%)
Does a family member/friend provide care for you?	Yes	145 (32.1%)	81 (30.8%)	64 (33.9%)
	No	307 (67.9%)	182 (69.2%)	135 (66.1%)

Bold values indicate statistical significance.

### Summarising index scores

- The mean *Independence score* was 11.7 ± 13.5 (median: 8; range: 0–83). The highest observed score was 83, but only 12 participants scored above 50 (i.e., above 50% of the maximum), while 91 individuals scored 0, indicating full independence.
- The mean *Risk of breakdown in care* was 3.1 ± 2.4 (median: 3; range: 0–12). Only one participant achieved the maximum score of 12, and just 49 individuals scored above 6 (over 50% of the maximum), while 47 had a score of 0.
- The mean *Risk of falls* was 1.8 ± 1.7 (median: 1; range: 0–8). A score of at least 3, indicating increased risk of falls, was observed in 140 participants (30.4%).

Table 3 presents descriptive statistics, Kruskal–Wallis test results and effect sizes for the EC summarising indices.

**Table 3:** Descriptive statistics, Kruskal–Wallis test results, and effect sizes (CI–confidence interval, Q1, Q3—lower and upper quartiles, H—Kruskal–Wallis test value, *η*^2^—effect size).

Index	Mean ± SD	95%CI	Median	Q1–Q3	H	*p*	*η*^2^
Independence score	3.71 ± 3.40	3.31–4.12	3.0	0.0–7.0	341.76	**<0.0001**	0.76
	42.63 ± 13.30	38.81–46.45	38.0	33.0–47.0			
	17.00 ± 4.71	16.18–17.82	16.0	13.0–20.0			
Risk of breakdown in care	2.14 ± 1.81	1.93–2.36	2.0	1.0–3.0	142.96	**<0.0001**	0.31
	7.16 ± 2.20	6.53–7.80	7.0	5.0–9.0			
	3.56 ± 1.86	3.24–3.89	3.0	2.0–5.0			
Risk of falls	1.02 ± 1.09	0.89–1.15	1.0	0.0–2.0	167.97	**<0.0001**	0.37
	4.20 ± 1.15	3.87–4.54	4.0	3.0–5.0			
	2.60 ± 1.64	2.32–2.89	2.5	1.0–4.0			

Bold values indicate statistical significance.

All summarising indices yielded statistical significance and a large effect.

### Cluster analysis

Three clusters were identified based on the three summarising indices:

*Cluster 1* (*n* = 275)—low needs

- *Lowest scores* on all indices.
- Mean *Independence score:* 3.7 ± 3.4 (median: 1; range: 0–3). All participants with a score of 0 were in this cluster.
- Mean *Risk of breakdown in care:* 2.1 ± 1.8 (median: 2; range: 1–3). Forty-four individuals with a score of 0 (93.6% of all with 0) were in this cluster.
- Mean *Risk of falls:* 1.0 ± 1.1 (median: 0; range: 0–2). Only 31 individuals (11.3%) had a *Risk of falls* score above 2 (increased risk).

*Cluster 2* (*n* = 49)—high needs

- *Highest scores* on all indices.
- Mean *Independence score:* 42.6 ± 13.3 (median: 38; range: 32.5–49). All participants with scores above 50% of the maximum were in this cluster.
- Mean *Risk of breakdown in care:* 7.2 ± 2.2 (median: 7; range: 5–9).
- Mean *Risk of falls:* 4.2 ± 1.2 (median: 4; range: 3–5). Forty-five individuals (91.8%) had a *Risk of falls* score above 2.

*Cluster 3* (*n* = 128)—medium needs

- *Intermediate scores* on all indices.
- Mean Independence score: 17.0 ± 4.7 (median: 16; range: 13–20).
- Mean Risk of breakdown in care: 3.6 ± 1.9 (median: 3; range: 2–5).
- Mean Risk of falls: 2.6 ± 1.6 (median: 2.5; range: 1–4). Half of the individuals (*n* = 64) had a Risk of falls score above 2.

**Figure 3:**
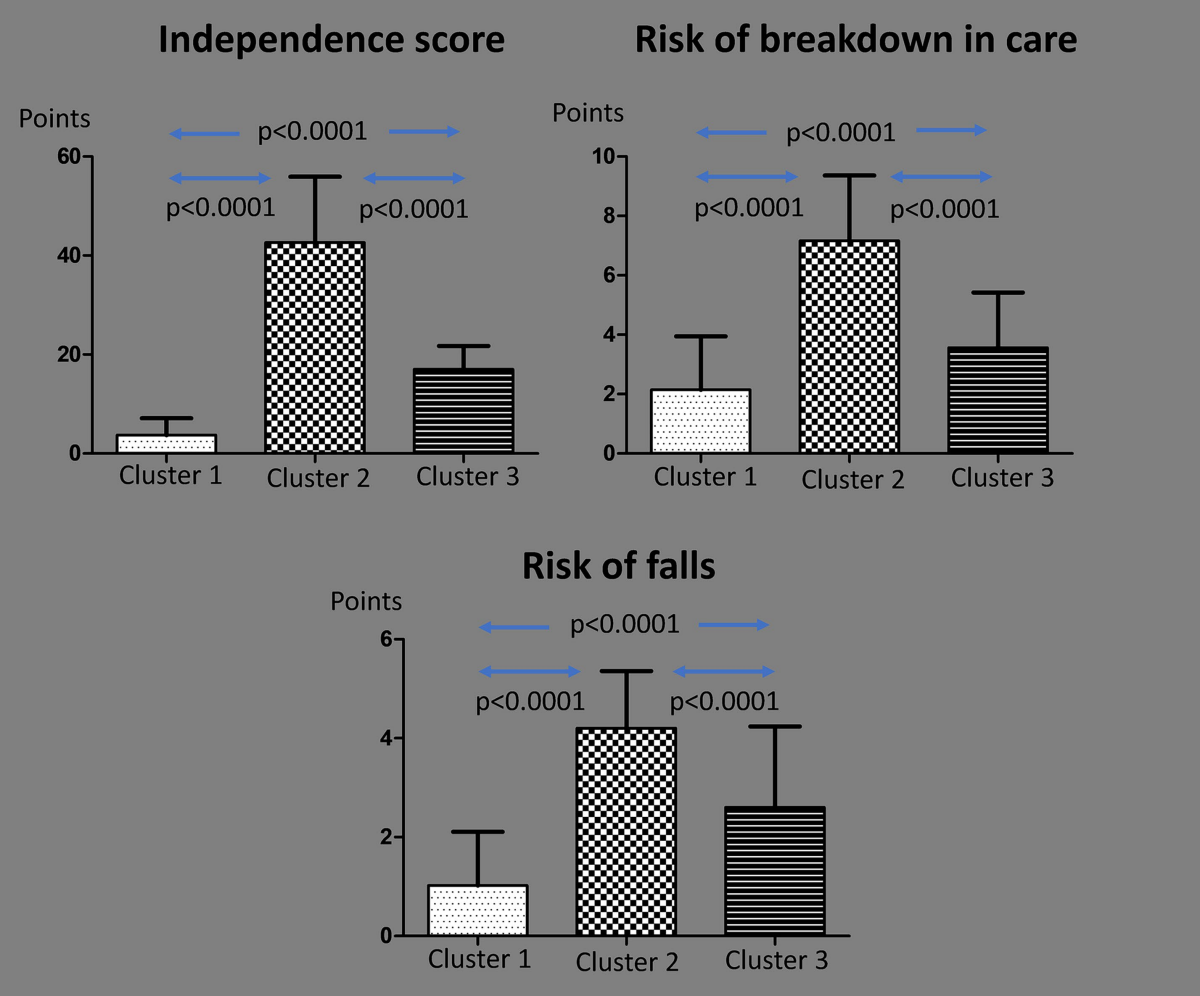
Comparison of mean summarising index scores by cluster.

A comparison of the summarising index scores across clusters is presented in Figure 3.

### Needs by domain

Comparison of clusters by the percentage of subjects with needs in each domain revealed significant differences across all domains except domains 5 (Accommodation and finances—this domain comprises three questions: Place of residence, Financial situation and Advice about financial allowances or benefits) and 6 (Staying healthy) (Table 4). Significant differences between clusters were also observed in the number of needs reported in each domain (Table 5).

**Table 4:** Mean number of needs indicated by the participants (mean ± SD; median; Q1–Q3) in each domain of the EASYCare 2010 Standard Questionnaire by cluster.

Domain (max number of needs)	Number of needs (mean ± SD; median; Q1–Q3)		
	Cluster 1 (C1)	Cluster 2 (C2)	Cluster 3 (C3)
I (4)	0.5 ± 0.9 (0; 0–1)	2.2 ± 1.1 (2; 1–3) ***p* < 0.001 vs C1**	1.3 ± 1.3 (1; 0–2) ***p* < 0.001 vs C1** ***p* < 0.001 vs C2**
II (13)	1.0 ± 1.0 (1; 0–2)	7.7 ± 1.9 (7; 7–8) ***p* < 0.001 vs C1**	3.3 ± 1.5 (3; 2–4) ***p* < 0.001 vs C1** ***p* < 0.001 vs C2**
III (8)	0.8 ± 1.1 (0; 0–1)	5.3 ± 1.5 (5; 4–7) ***p* < 0.001 vs C1**	2.5 ± 1.7 (2; 1–4) ***p* < 0.001 vs C1** ***p* < 0.001 vs C2**
IV (5)	0.6 ± 1.0 (0; 0–1)	1.6 ± 1.1 (1;1–2) ***p <* 0.001 vs C1**	1.6 ± 1.4 (1; 0–3) ***p* < 0.001 vs C1**
V (3)	0.5 ± 0.6 (0; 0–1)	1.1 ± 1.1 (1;0–2) ***p* < 0.01 vs C1**	0.9 ± 1.1 (0; 0–2) ***p <* 0.01 vs C1**
VI (7)	2.1 ± 1.4 (2; 1–3)	3.2 ± 1.4 (3; 2–4) ***p* < 0.001 vs C1**	2.8 ± 1.4 (3; 2–4) ***p <* 0.001 vs C1**
VII (9)	2.1 ± 1.9 (2; 1–3)	4.5 ± 2.3 (5; 2.5–6.5) ***p* < 0.001 vs C1**	3.0 ± 1.9 (3; 2–4) ***p <* 0.001 vs C1** ***p <* 0.01 vs C1**
Total	7.7 ± 4.0 (7; 5–11)	25.5 ± 5.3 (26; 21–25.5) ***p* < 0.001 vs C1**	15.4 ± 5.3 (15; 12–19) ***p <* 0.001 vs C1** **p *<* 0.001 vs C2**

Bold values indicate statistical significance.

**Table 5:** Number (%) of participants reporting needs in each domain by cluster.

Domain	Number of people with needs (percentage)			Statistical analysis
	Cluster 1	Cluster 2	Cluster 3	
I	101 (36.7%)	47 (95.9%)	81 (63.3%)	***p* < 0.001**
II	179 (65.1%)	49 (100.0%)	127 (99.2%)	***p* < 0.001**
III	137 (49.8%)	49 (100.0%)	111 (86.7%)	***p* < 0.001**
IV	97 (35.3%)	41 (83.7%)	92 (71.9%)	***p* < 0.001**
V	116 (42.2%)	27 (55.1%)	63 (49.2%)	*p* = 0.1530
VI	244 (88.7%)	47 (95.9%)	121 (94.5%)	*p* = 0.0744
VII	209 (76.0%)	48 (98.0%)	121 (94.5%)	***p* < 0.001**

Bold values indicate statistical significance.

Table 6 presents descriptive statistics, Kruskal–Wallis test results and effect sizes for the individual EC domains.

**Table 6:** Descriptive statistics, Kruskal–Wallis test results, and effect sizes (CI—confidence interval, Q1, Q3—lower and upper quartiles, H—Kruskal–Wallis test value, η^2^—effect size).

Domain	Mean ± SD	95%CI	Median	Q1–Q3	H	*p*	η^2^
I	0.53 ± 0.87	0.43–0.63	0.0	0.0–1.0	104.73	**<0.0001**	0.23
	2.22 ± 1.09	1.91–2.54	2.0	1.0–3.0			
	1.33 ± 1.27	1.11–1.55	1.0	0.0–2.0			
II	1.03 ± 0.97	0.92–1.15	1.0	0.0–2.0	261.40	**<0.0001**	0.58
	7.67 ± 1.94	7.12–8.23	7.0	7.0–8.0			
	3.28 ± 1.49	3.02–3.54	3.0	2.0–4.0			
III	0.83 ± 1.06	0.70–0.95	0.0	0.0–1.0	194.11	**<0.0001**	0.43
	5.29 ± 1.50	4.85–5.72	5.0	4.0–7.0			
	2.46 ± 1.71	2.16–2.76	2.0	1.0–4.0			
IV	0.61 ± 0.98	0.49–0.72	0.0	0.0–1.0	75.63	**<0.0001**	0.16
	1.59 ± 1.14	1.27–1.92	1.0	1.0–2.0			
	1.56 ± 1.37	1.32–1.80	1.0	0.0–3.0			
V	0.47 ± 0.63	0.40–0.55	0.0	0.0–1.0	15.39	**0.0005**	0.03
	1.06 ± 1.14	0.73–1.39	1.0	0.0–2.0			
	0.91 ± 1.11	0.71–1.10	0.0	0.0–2.0			
VI	2.07 ± 1.38	1.90–2.23	2.0	1.0–3.0	39.98	**<0.0001**	0.08
	3.16 ± 1.40	2.76–3.57	3.0	2.0–4.0			
	2.84 ± 1.44	2.58–3.09	3.0	2.0–4.0			
VII	2.13 ± 1.89	1.91–2.36	2.0	1.0–3.0	53.33	**<0.0001**	0.11
	4.51 ± 2.29	3.85–5.17	5.0	3.0–6.0			
	3.03 ± 1.89	2.70–3.36	3.0	2.0–4.0			
Total	7.67 ± 3.96	7.20–8.14	7.0	5.0–11.0	241.90	**<0.0001**	0.53
	25.51 ± 5.29	23.99–27.03	26.0	21.0–29.0			
	15.41 ± 5.32	14.48–16.34	15.0	12.0–19.0			

Bold values indicate statistical significance.

All domains yielded statistical significance; the effect was large for the domains I-IV and moderate for domains V-VII.

### Sociodemographic differences between clusters

Significant differences in educational attainment were observed between clusters. The proportion of individuals with only primary education was significantly lower in Cluster 1 compared to Cluster 2 (20.7% vs. 46.9%; *p* < 0.001) and Cluster 3 (33.6%; *p* < 0.01). Moreover, the proportion with higher education was greater in Cluster 1 than in Cluster 2 (38.9% vs. 20.4%; *p* < 0.05). No differences were observed for secondary education. Additionally, the frequency of having a caregiver was significantly lower in Cluster 1 compared to both Cluster 2 (26.0% vs. 46.0%; *p* < 0.01) and Cluster 3 (26.0% vs. 37.5%; *p* < 0.05). No significant differences were found for the other sociodemographic variables analysed.

### Multivariate logistic regression analysis

Tables 7–9 present the results of multivariate analysis of Clusters 1–3. Clusters (C1, C2, C3) constituted the independent variable; the dependent variables were: sex, age, residence area, marital status, living arrangements, education, caregiver presence and financial situation.

**Table 7:** The results of the multivariate logistic regression analysis for Cluster 1.

Variable	Regression coefficient	Standard error	Wald statistic	*p*-value	Odds ratio	−95% CI	+95% CI
Constant term	−0.493	0.567	0.75	0.3854	0.611	0.201	1.858
Sex	0.226	0.205	1.21	0.2708	1.253	0.839	1.872
Age	−0.045	0.241	0.03	0.8537	0.956	0.596	1.535
Residence area	0.458	0.531	0.74	0.3888	1.580	0.558	4.473
Marital status	−0.032	0.324	0.01	0.9206	0.968	0.513	1.828
Living arrangements (2)	0.277	0.363	0.58	0.4448	1.319	0.648	2.686
Living arrangements (3)	−0.253	0.339	0.56	0.4549	0.776	0.400	1.508
Education (2)	0.803	0.260	9.52	**0.0020**	2.232	1.340	3.716
Education (3)	0.953	0.263	13.13	**0.0003**	2.594	1.549	4.344
Caregiver presence	−0.458	0.214	4.56	**0.0327**	0.633	0.416	0.963
Financial situation	−0.029	0.222	0.02	0.8971	0.972	0.628	1.503

Bold values indicate statistical significance.

**Table 8:** The results of the multivariate logistic regression analysis for Cluster 2.

Variable	Regression coefficient	Standard error	Wald statistic	*p*-value	Odds ratio	−95% CI	+95% CI
Constant term	−2.809	1.134	6.14	0.0132	0.060	0.007	0.556
Sex	−0.525	0.319	2.71	0.0994	0.592	0.317	1.105
Age	0.433	0.353	1.51	0.2198	1.542	0.772	3.078
Residence area	1.105	1.103	1.00	0.3165	3.018	0.348	26.202
Marital status	0.719	0.534	1.81	0.1784	2.052	0.720	5.844
Living arrangements (2)	−0.784	0.534	2.16	0.1421	0.457	0.160	1.300
Living arrangements (3)	−0.633	0.505	1.57	0.2103	0.531	0.197	1.430
Education (2)	−0.853	0.381	5.00	**0.0253**	0.426	0.202	0.900
Education (3)	−1.278	0.423	9.12	**0.0025**	0.279	0.122	0.638
Caregiver presence	0.567	0.323	3.07	0.0797	1.762	0.935	3.321
Financial situation	0.293	0.361	0.66	0.4167	1.341	0.661	2.722

Bold values indicate statistical significance.

**Table 9:** The results of the multivariate logistic regression analysis for Cluster 3.

Variable	Regression coefficient	Standard error	Wald statistic	*p*-value	Odds ratio	−95% CI	+95% CI
Constant term	0.148	0.564	0.07	0.7934	1.159	0.384	3.502
Sex	−0.020	0.219	0.01	0.9282	0.980	0.638	1.506
Age	−0.178	0.261	0.47	0.4943	0.837	0.502	1.395
Residence area	−0.865	0.529	2.68	0.1018	0.421	0.149	1.187
Marital status	−0.283	0.339	0.70	0.4043	0.754	0.387	1.465
Living arrangements (2)	0.068	0.398	0.03	0.8635	1.071	0.490	2.338
Living arrangements (3)	0.602	0.368	2.67	0.1021	1.826	0.887	3.757
Education (2)	−0.459	0.275	2.78	0.0955	0.632	0.369	1.084
Education (3)	−0.482	0.277	3.02	0.0824	0.618	0.359	1.064
Caregiver presence	0.246	0.228	1.16	0.2814	1.278	0.818	1.999
Financial situation	−0.085	0.235	0.13	0.7189	0.919	0.580	1.457

Bold values indicate statistical significance.

For Cluster 1, the model was statistically significant (*p* = 0.0013), though the overall model fit was modest: Pseudo *R*^2^ = 0.048, Nagelkerke *R*^2^ = 0.084, Cox–Snell *R*^2^ = 0.062. Variables significantly associated with membership in Cluster 1 included:

- Education—Secondary (*p* = 0.0020): individuals with secondary education were more than 2.23 times more likely to belong to C1 than those with primary education.
- Education—Higher (*p* = 0.0003): individuals with higher education were more than 2.59 times more likely to belong to C1 than those with primary education.
- Having a caregiver (*p* = 0.0327): individuals with a caregiver were approximately 1.58 times less likely to belong to C1 than those without a caregiver.

The model for Cluster 2 was statistically significant (*p* = 0.0111), although the model fit was modest: Pseudo *R*^2^ = 0.074, Nagelkerke *R*^2^ = 0.099, and Cox–Snell *R*^2^ = 0.049. The variables notably associated with membership in Cluster 2 (C2) were as follows:

- Education—Secondary (*p* = 0.0253): individuals with secondary education were nearly 2.35 times less likely to belong to C2 than those with primary education.
- Education—Higher education (*p* = 0.0025): individuals with higher education were more than 3.59 times more likely to be classified in C2 than those with primary education.

These findings demonstrate that education level significantly influences the probability of belonging to Cluster 2, though the overall model explains only a modest proportion of variance.

The model for Cluster 3 was not statistically significant (*p* = 0.1777). No variables demonstrated statistical significance (*p* > 0.05), indicating that none of the factors included were associated with membership in Cluster 3.

## Discussion

A comprehensive assessment of older adults’ health and social needs is fundamental for informing tailored interventions that support ageing in place. Such investigations have not been conducted in Central Asian countries; accordingly, we undertook this research using the validated Russian-language version of the EASYCare questionnaire.

Our previous study has focused on Kazakh-speaking populations in Kazakhstan (16). However, given that Russian is an official language alongside Kazakh (10), and that a considerable portion of the older population may not be proficient in Kazakh, additional analyses were warranted. It is important to note that answering questions accurately in a language other than one’s mother tongue can be challenging, particularly when that language is not used in daily life (25). As Lundmark et al. (22) noted, language barriers are a significant factor contributing to nonresponse bias in survey research. There is a recognised need for the translation of research instruments in cross-national and multilingual surveys, especially in countries where several languages are spoken (26). Researchers often assume that all individuals can communicate in the official language and understand the questions to a sufficient degree; however, Sarac and Koc (25) have demonstrated that this is not always the case. Indeed, evidence suggests that, where possible, questionnaires should be administered in respondents’ native language to optimise accessibility and comprehension (27). The use of a Russian-language version of the questionnaire in this study ensured that participants could respond in their preferred and most familiar language, thereby enhancing the reliability and validity of the data collected.

Our cluster analysis identified three distinct groups of older adults, discriminated primarily by varying levels of health and social needs. Education constituted the most salient distinguishing variable between clusters; the group exhibiting the fewest unmet needs (Cluster 1) had the highest proportion of individuals with higher education, whereas the group with the greatest number of needs (Cluster 2) comprised predominantly those with only primary education. This finding is consonant with earlier research demonstrating that education positively influences access to health services and understanding health issues (28) and is an independent predictor of a more positive self-assessment of successful ageing (29). Higher education levels have also been found to be linked with better mental health state (30). Furthermore, educational attainment is strongly associated with health literacy (31–33). It operates as a key social determinant of health by enhancing patient-physician communication, particularly in bridging medical terminology and lay understanding (34). Education empowers older adults to seek, appraise, and effectively use health information (35). Educational initiatives, especially those targeting individuals of lower educational status, have demonstrated effectiveness in enhancing health literacy and self-management skills within rural Kazakh populations (36).

The limited sense of agency among patients regarding their health is a pervasive challenge in Kazakhstan; medical professionals are commonly considered the only authorities in managing the well-being (37). For a sustainable and effective healthcare system, conceptual shifts are needed both towards patient empowerment (38) and away from a disease-focused approach towards models grounded in long-term personal relationships and social context (39). Achieving sustainable development further necessitates the involvement of a broader spectrum of professionals other than doctors to promote health literacy, particularly among those with only primary education (40). In neighbouring Kyrgyzstan, the involvement of trained volunteers in communities and primary care facilities improved health-related knowledge and behaviours (41). The role of nurses in primary healthcare is discussed in Kazakhstan, as one involving active participation in diagnostic and treatment processes (40) and screening programmes (42). It is also noteworthy that, according to some researchers, a social worker may act as an interpreter and mediator, serving as a bridge to address communication gaps between patients, families, and other members of the therapeutic team (43). Recent studies relating to cardiovascular diseases in Kazakhstan also underscore the need for a strengthened health literacy agenda within primary care (44), thus reinforcing the relevance of our findings.

Beyond education, we observed that individuals in the cluster with the lowest indices (Cluster 1) less frequently reported having a caregiver compared to their higher-need counterparts. While it may be anticipated that individuals with greater needs require support, the ECQ instrument determines unmet—not met—needs in the first place. This distinction suggests that discordances may exist between the self-assessed needs of older adults and those identified by their caregivers, a phenomenon documented in previous research (45, 46). The association between having a caregiver and a greater number of unmet needs may be indicative of inadequate caregiver preparedness. Notably, caregiver skills assessments in eastern Kazakhstan have highlighted insufficient training and resultant barriers to effective care, particularly among those providing support post-stroke (47). Unmet needs may also be attributed to caregiver burden and burnout—a significant risk in Kazakhstan, where cultural traditions assign primary responsibility for eldercare to adult children, most frequently daughters (13, 48, 49). This gendered responsibility is further complicated by the phenomenon of ‘sandwiched’ caregivers, who concurrently care for both their own children and ageing parents, engendering additional stress (50).

Societal shifts towards individualisation and family nuclearisation are altering the living arrangements for Kazakhstan’s older people, resulting in increased proportions of those living alone or with only a spouse, as substantiated by our findings (nearly one in five older individuals surveyed lived alone, and almost one in three lived only with a spouse) *and* by recent demographic analyses (51). Given these trends and the overarching goal of sustainable development to address societal needs and aspirations (52), it is imperative to strengthen the availability of formal care resources for older adults and to bolster support for informal caregivers through targeted education and needs assessment programmes for both caregivers and caretakers (9). Our study contributes to this discourse by foregrounding older adults’ self-reported needs, highlighting the scope of unmet needs and examining their correlates.

### Limitations

Our study is not without limitations. Using a non-random sample with an over-representation of urban residents, excluding individuals with cognitive impairment, and data collection occurring during the COVID-19 pandemic may restrict the generalisability of findings (older adults constituted a group that faced particular challenges and increased mortality at that time (53, 54)). Additionally, the cross-sectional study design precludes assessment of causal inference. However, the study’s main strengths include a relatively large, multicentre sample and the application of a validated instrument for needs assessment. Importantly, our findings elucidate potentially modifiable correlates of unmet need, supporting the rationale for tailored interventions. Such initiatives gain further urgency in the context of demographic changes in Kazakhstan, as the population ages.

### Conclusion

The study identifies a clear association between unmet needs among older adults and low educational attainment, simultaneously highlighting the pivotal, yet often under-supported, role of informal caregivers. These insights establish a foundation for understanding the needs of older adults and provide a baseline for subsequent policy and planning activity. As Kazakhstan’s demographic profile evolves, proactive strategies to design age-friendly healthcare and social services must recognise intra-group heterogeneity within the older population. By doing so, both policymakers and healthcare providers can allocate resources more efficiently and design tailored interventions that address the specific needs of various subgroups, thereby enhancing functional independence for Kazakhstan’s ageing citizenry.

## Data Availability

The raw data supporting the conclusions of this article will be made available by the authors, without undue reservation.
